# Identification of circular RNAs expression pattern in caprine fetal fibroblast cells exposed to a chronic non-cytotoxic dose of graphene oxide-silver nanoparticle nanocomposites

**DOI:** 10.3389/fbioe.2023.1090814

**Published:** 2023-03-16

**Authors:** Yu-Guo Yuan, Yi-Tian Xing, Song-Zi Liu, Ling Li, Abu Musa Md Talimur Reza, He-Qing Cai, Jia-Lin Wang, Pengfei Wu, Ping Zhong, Il-Keun Kong

**Affiliations:** ^1^ College of Veterinary Medicine, Yangzhou University, Yangzhou, China; ^2^ Jiangsu Co-Innovation Center of Prevention and Control of Important Animal Infectious Diseases and Zoonoses, Yangzhou University, Yangzhou, China; ^3^ Department of Molecular Biology and Genetics, Faculty of Basic Sciences, Gebze Technical University, Gebze, Kocaeli, Türkiye; ^4^ Division of Applied Life Science (BK21 Four), Institute of Agriculture and Life Science, Gyeongsang National University, Jinju, Gyeongnam, Republic of Korea

**Keywords:** nanotoxicity, circRNA, reduced graphene oxide, silver nanoparticles, caprine fetal fibroblast cells

## Abstract

The widespread use of graphene oxide-silver nanoparticle nanocomposites (GO-AgNPs) in biomedical sciences is increasing the chances of human and animal exposure to its chronic non-toxic doses. Exposure to AgNPs-related nanomaterials may result in the negative effect on the dam, fetus and offspring. However, there are only little available information for profound understanding of the epigenetic alteration in the cells and animals caused by low-dose chronic exposure of GO-AgNPs. The present study investigated the effect of 0.5 μg/mL GO-AgNPs for 10 weeks on the differential expression of circular RNAs (circRNAs) in caprine fetal fibroblast cells (CFFCs), and this dose of GO-AgNPs did not affect cell viability and ROS level. We predicted the functions of those differentially expressed (DE) circRNAs in CFFCs by bioinformatics analysis. Furthermore, we validated the expression of ten DE circRNAs using quantitative real-time reverse transcription-polymerase chain reaction (qRT-PCR) to ensure the reliability of the sequencing data. Our results showed that the DE circRNAs may potentially regulate the GO-AgNPs-inducing epigenetic toxicity through a regulatory network consisted of circRNAs, miRNAs and messenger RNAs (mRNAs). Therefore, the epigenetics toxicity is essential to assess the biosafety level of GO-AgNPs.

## Introduction

The potential therapeutic and biomedical use of nanotechnology is not limited to the treatment of human diseases, it also has an enormous potential to be applied in veterinary biomedical sciences. Take an example, the recent integration of nanotechnology in different aspects of plant and animal production such as diagnosis of diseases, treatment, vaccination, reproduction, feeding, and hygienic management (Chariou et al., 2020; Kutawa et al., 2021). Researches have shown that graphene/silver nanocomposite (generated by coupling of silver nanoparticle nanocomposites (AgNPs) and graphene-based nanomaterials) has enhanced its antibacterial activity with the combination of therapeutic strategies (Tan et al., 2020; Porter et al., 2021). Besides, there are other issues that are being addressed by the practical applications of silver-nanoparticle-based materials, such as overcoming the challenges of antibiotic resistance and enhancing the overall veterinary practices (Yuan et al., 2017a; Estevez et al., 2021). This widespread use of nanotechnology, in particular, the use of graphene/silver nanocomposite has raised the concern of its potential effects on human health. Thus, a comprehensive analysis is required to know the possible long- and short-term effects of AgNP-related materials before its full-scale application in veterinary and animal sciences (Ema et al., 2017a; Yuan et al., 2021). A number of previous studies were conducted to evaluate possible mechanisms of graphene oxide or AgNPs-induced toxicity with various kinds of cells lines and animal models (Liao et al., 2019; Ghulam et al., 2022). Numerous studies have also shown the toxicological effects of graphene oxide-silver nanoparticle nanocomposites (GO-AgNPs) on human cells and animal (Ali et al., 2018; Courtois et al., 2019; Yuan et al., 2021), which even remains two generations post-maternal exposure (Ellis et al., 2020). These studies have identified distinctive cellular responses including long-term stress response, substantial modifications in the regulation of genes, and an upregulation of EGF signaling. However, it is not practical to correlate the findings of the acute *in vitro* nanoparticles exposure to the chronic *in vitro/vivo* model (Comfort et al., 2014). For example, chronic prenatal exposure to AgNPs may compromise the neonatal and postnatal development without showing any visible effect on the parents (Zhang et al., 2015; Becaro et al., 2021). Therefore, the potential reproductive and developmental toxicity in livestock is a valid concern related to the practical use of GO-AgNPs (Yuan et al., 2021), and indeed required to be investigated further (Ema et al., 2017b; Becaro et al., 2021; Wang et al., 2018; Dugershaw et al., 2020). As different cell types show varying sensitivity to nanoparticle, it is important to use varieties of cell types to get reliable observations. In our previous study, we have used caprine fetal fibroblast cells (CFFCs), which is considered as a suitable *in vitro* model for studying long term effect of the nanomaterial-mediated toxicity on the fetus (Yuan et al., 2021).

In the past, AgNPs or GO-AgNPs mediated toxicity has been studied focusing its impacts on the genetic and biological alterations, but recently nanoparticle mediated epigenetic toxicity becomes an attractive field of research (Gedda et al., 2019). The regulatory roles of epigenetic factors on cellular and biological processes are well evident, these factors include DNA methylation and hydroxymethylation, histone modification, chromatin remodeling, RNA methylation, and different non-coding RNAs such as microRNA (miRNA), transfer RNA (tRNA), long non-coding RNAs (lncRNAs) and circular RNAs (circRNAs). Recent research has identified that exposure of mammalian cells to AgNPs or GO-AgNPs has changed its DNA methylation, post-translational histone modifications, and microRNAs expression (Zhang et al., 2015; Zhang et al., 2020; Yuan et al., 2021). However, the expression pattern of circRNAs in AgNPs or GO-AgNPs-induced toxicity remains unexplored. CircRNA is a kind of ncRNA, whose structure comprises covalently closed loops without a 5′ end cap and 3′ end poly (A) tail. The majority of circRNAs are conserved across different species, however, it shows tissue or developmental-stage-specific expression (Memczak et al., 2013; Kristensen et al., 2019). The circRNAs are functionally different from miRNA, piRNA and lncRNA, and play an important role as microRNA sponges, regulating gene splicing and transcription, as well as RNA-binding protein sponges and protein/peptide translating (Patop et al., 2019). It also showed close involvement in a series of physiological processes that are regulating fetal growth, muscle formation, and lactation in mammals (Huang et al., 2020; He et al., 2021). CircRNAs are also found to be important in the regulation of post-transcriptional gene expression (Hansen et al., 2013).

Our previous study demonstrated that GO-AgNPs for 48 h can incite DNA hypomethylation, result in the production of reactive oxygen species (ROS) and apoptosis in caprine fetal fibroblast cells (Yuan et al., 2021). Since the synthesized GO-AgNPs is a potential antibacterial agent in veterinary medicine for the treatment of bovine and caprine mastitis during pregnancy, which take long time for treatment. Though developmental effects have been examined at non-maternal toxic doses to assess the direct effects on the embryo/fetus, the epigenetic alterations of which are not clear. The role of epigenetic modifications of circRNAs in GO-AgNPs’ toxicity remains unexplored. Thus, we suppose that circRNAs expression pattern may change in GO-AgNPs-treated cells. This study aims to evaluate the chronic *in vitro* effect of low concentration GO-AgNPs (0.5 μg/mL) on the circRNAs expression pattern in CFFCs. We have explored the expression pattern of both circRNAs and messenger RNAs (mRNAs) in CFFCs after 10-week of GO-AgNPs exposure and predicted a regulatory network (circRNAs-miRNA-mRNA) that potentially regulates the GO-AgNPs mediated epigenetic toxicity in CFFCs.

## Materials and methods

### Cell culture and chemical treatment

CFFCs that were isolated from caprine fetus and stored in our lab (Yuan et al., 2021). The goat owners were informed as to the purpose of this study and provided informed consent for their animals to be used in this study. CFFCs were recovered by dipping and continuous shaking the cryovial in 37°C water bath ∼1 min and seeded into 6-well plate containing 10% FBS-DMEM/F-12 (Thermo Fisher Scientifc, Waltham, MA, United States) supplemented with penicillin (100 units/mL), and streptomycin (100 μg/mL). The cells were cultured at 37°C in a humidified atmosphere containing 5% CO_2_ as described previously (Yuan et al., 2021). GO-AgNPs that was prepared and stored in our lab was sonicated for 20 min before use (Yuan and Gurunathan, 2017b). The viability and ROS level of CFFCs were not significantly reduced with the concentration of 1 μg/mL GO-AgNPs (Yuan et al., 2021). The practical applications of silver-nanoparticle-based materials to overcome antimicrobial resistance in goat and cow mastitis will be taken for more than some weeks. Therefore, the cells were exposed to continuous treatment with GO-AgNPs (0 and 0.5 μg/mL) for 10 weeks in 10% FBS-DMEM/F-12. During the treatment period, the cells were passaged every 2–3 days.

### Cell viability assay

The cell viability of GO-AgNPs treated and control cells was measured every week using the cell counting kit-8 (CCK-8; Rockville, MD, United States) according to the manufacturer’s instruction as described previously (Yuan et al., 2021). In brief, at the end of treatment period, 10 μL CCK-8 was added into each well containing cells with 100 μL medium and incubated for 30 min at 37°C in dark. The absorbance at 450 nm was measured using a microplate reader (BioTek Synergy 2, United States).

### Measurement of ROS production

Dichlorodihydrofluorescein diacetate (DCFH-DA) was used to detect intracellular ROS induced by 0 and 0.5 μg/mL of GO-AgNPs according to the manufacturer’s instructions as described previously (Yuan et al., 2021). In brief, CFFCs were incubated with 10 μM DCFH-DA for 30 min at 37°C, then were rinsed twice with PBS and harvested using 0.25% Trypsin-EDTA. The intracellular ROS accumulation was measured by flow cytometry (Beckman-Coulter, United States).

### Extraction and preparation of RNA samples

RNA samples were prepared from the cells using Trizol (TANGEN, Beijing, China) according to the manufacturer’s protocol and the optical density (OD260/280) value of the RNA concentration was measured with a NanoDrop ND-2000 instrument (Thermo Fisher Scientific, Waltham, MA, United States). Finally, the integrity of the isolated RNA was confirmed by agarose gel electrophoresis.

### High-throughput sequencing

High-throughput transcriptome sequencing and bioinformatics analysis were performed using the commercial service provided by Cloud-Seq Biotech (Shanghai, China). In brief, the ribosomal RNA (rRNA) was depleted by treating the total RNA with a Ribo-Zero rRNA Removal kit (Illumina, San Diego, CA, United States). The purified RNA samples were used to generate the RNA library using the TruSeq Stranded Total RNA Library Prep kit (Illumina, San Diego, CA, United States) according to the manufacturer’s protocol. The quantification of the constructed libraries as well as their quality was measured using BioAnalyzer 2100 system. Finally, 10-pM libraries were denatured into single-stranded DNA molecules, captured on Illumina flow cells, amplified *in situ*, clustered, and 150 cycles of sequencing were performed on the Illumina HiSeq sequencer according to the manufacturer’s instructions.

### CircRNA sequencing analysis

The paired terminal readings were obtained from the Illumina HiSeq 4,000 sequencer, and Q30 was used to performed quality control. Cutadapt software (version 1.9.3) were used to primarily screened High-quality reads, and the low-quality reads were removed after performing 3′ adapter trimming. STAR software (version 2.5.1b) was used to map and align high-quality reads with the reference genome/transcriptome. After that, some nucleotide sequences from the reads were selected as anchor points and the results were inputted into the DCC software (version 0.4.4) to compare the connected and unconnected reads for identifying possible circRNAs. Finally, the data were normalized using the EdgeR software (version 3.16.5) and analyzed the identified DE circRNAs.

### Analysis of DE circRNAs and mRNA

The DE circRNAs and mRNAs between the control and 0.5 μg/mL of GO-AgNPs groups was calculated using standardized readings. CircRNAs and mRNAs with a fold change of ≥ 2.0 and *p* < 0.05 were considered as DE as well as statistically significant.

### Gene ontology (GO) and kyoto encyclopedia of genes and genomes pathway analyses

GO (http://www.geneontology.org) and KEGG (http://www.genome.jp/kegg) were used to analyze genes that are related to the DE circRNAs and mRNA. GO analysis is divided into three aspects: molecular function (MF), biological process (BP), and cellular component (CC). The top 10 enriched GO terms were ranked according to the *p*-value. The possible biological functions of DE circRNA and mRNA were analyzed by KEGG pathway analysis.

### Validation of DE circRNAs

In order to verify the data obtained from high-throughput RNA sequencing, the expression of five upregulated circRNAs and five downregulated circRNAs was verified by qRT-PCR while the expression of GAPDH was considered as the internal control. In brief, total RNA was reverse-transcribed into complementary DNA using the PrimeScript RT kit (Perfect Real Time; Takara, Osaka, Japan), and qRT-PCR was performed with SYBR Premix Ex Taq II (TaKaRa) using the Roche LightCycler 480II PCR instrument (Basel, Switzerland) for circRNA analyses according to the manufacturer’s protocols. The independent experiments were performed three times and the expression was determined using a threshold cycle while the relative expression level was calculated using the 2^−ΔΔCT^ method. The primer sets that were used to amplify the selected circRNAs are enlisted in Table 1.

**TABLE 1:** Primer sequences for qRT-PCR validation of selected circRNAs.

CircBase ID	F/R	Primer sequence	Size (bp)
7:84193926-84207218+	F	TAG​ATA​GCT​TGC​GTC​GGA​CT	104
	R	GGA​GTT​GGC​AAA​ATA​CTT​GTC	
20:11474578-11474795−	F	TGA​CTT​AGC​CTC​TCA​TGG​T	112
	R	TAA​ACA​CAC​TCG​TAG​GCA​A	
20:2878853-2958684 +	F	TCT​TTA​ATG​GAT​ACC​GGA​T	156
	R	TTT​CCA​CGA​TGA​AAC​TTG​A	
19:36471752-36473897−	F	ATC​CTG​AAG​AAT​ATG​CAC​GAC	163
	R	TCC​AAG​TCA​TAC​TCG​CTC​GTC	
19:17783114-17803011+	F	TGG​GCA​GAC​GTT​AAC​ATT​CAG​A	174
	R	TAA​GCA​AGG​CAC​GTC​GAC​CA	
2974975273050017:9759064-9766303+	F	AAG​TCT​GGC​TCA​AGT​ATT​CGT	146
	R	TCT​GAG​CTC​CAT​CAT​GTC​ACT	
2:130861551-130862388+	F	CAC​GAG​GTT​AAG​CCC​ATC​CAC	146
	R	GTT​GCT​GGA​CAT​CAT​CAC​CAC	
8:90045854-90066768+	F	ACC​CTG​CAA​ATA​TAA​AGC​TG	109
	R	CCT​CCG​TAT​TTA​CAT​GAC​GA	
4:33600215-33618663+	F	GTT​CCA​TTG​ACA​ATT​TGC​CAG	100
	R	TCG​CAG​CAT​CTT​GAA​ACC​T	
11:29744772-29745680−	F	AAA​TCT​TCT​CGT​ATT​TGC​TGG​A	131
	R	CCG​GAT​TCT​TCT​GCA​ACC​AT	
GAPDH	F	CGT​TGC​CAT​CAA​TGA​CCC​CTT	
	R	ATT​GAT​GAC​GAG​CTT​CCC​GTT​C	

### Analysis of the circRNA–miRNA-mRNA network and related prediction

TargetScan (version 7.0) and MiRanda (version 3.3a) were used to predict the miRNA binding sites along with the target mRNAs. After that, Cytoscape software (version 3.1.0) was used to construct a network map of circRNA-miRNA-mRNA based on the results predicted before.

### Data analysis

Analysis of the experimental data was performed using the GraphPad Prism 7.0 (GraphPad Software Inc., CA, United States). Student’s *t*-test and one-way ANOVA were used to compare differences between groups as appropriate. Data are presented as the mean ± standard deviation (SD), and a *p*-value < 0.05 was considered as statistically significant. The data are representative of at least three experiments.

## Results

### Viability of the CFFCs upon chronic exposure to GO-AgNPs

**FIGURE 1:**
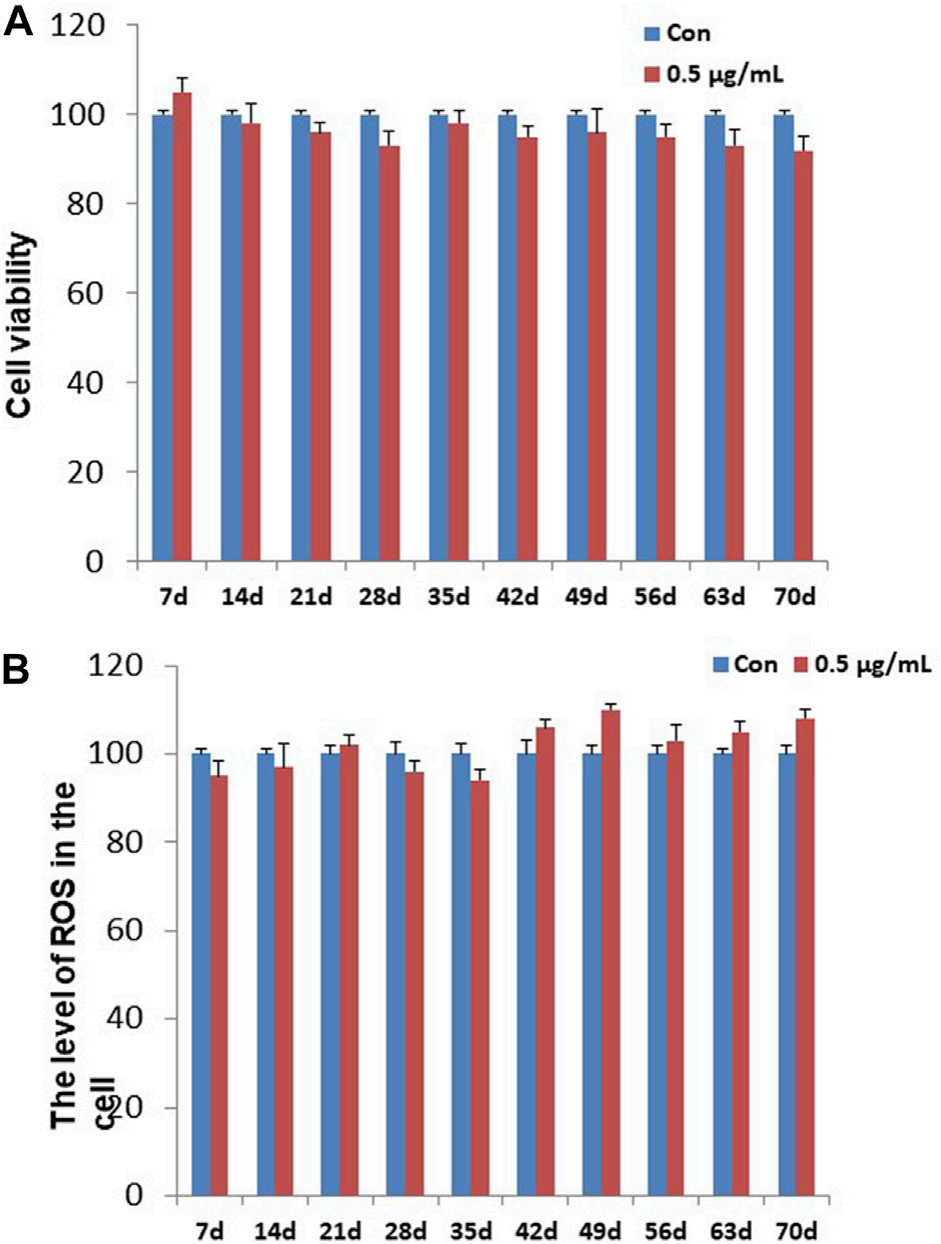
Effects of GO-AgNPs on the proliferation and ROS level of caprine fetal fibroblast cells (CFFCs). CFFCs were exposed to 0 and 0.5 μg/mL of GO-AgNPs for 70 days. **(A)** The percentage of CFFCs viability was then calculated relative to the control group (0 μg/mL). **(B)** Total reactive oxygen species (ROS) generation in GO-AgNP-treated cells. Caprine fetal fibro-blast cells (CFFCs) were checked for ROS every week. The percentage of ROS generation relative to the untreated control group (0 μg/mL). Values are presented as the mean ± SD of four independent experiments.

To determining the chronic cytotoxic effect of the synthesized GO-AgNPs, the CFFCs were cultured in the medium containing 0.5 μg/mL GO-AgNPs for 10 weeks. The viability of the cells was measured every week using CCK-8 kit. As shown in Figure 1A, treatment with 0.5 μg/mL GO-AgNPs for 10 weeks did not cause significant reduction in the viability of CFFCs compared to the non-treated cells.

### The level of intracellular ROS in GO-AgNPs-treated CFFCs

To detect the changes in ROS in the CFFCs that was exposed to the chronic dose of GO-AgNPs, intracellular ROS generation in both treated (0.5 μg/mL GO-AgNPs) and non-treated CFFCs was measured at every week. As shown in Figure 1B, treatment with 0.5 μg/mL GO-AgNPs for 10 weeks did not increase intracellular ROS production significantly.

### The detection of circRNAs in CFFCs

**FIGURE 2:**
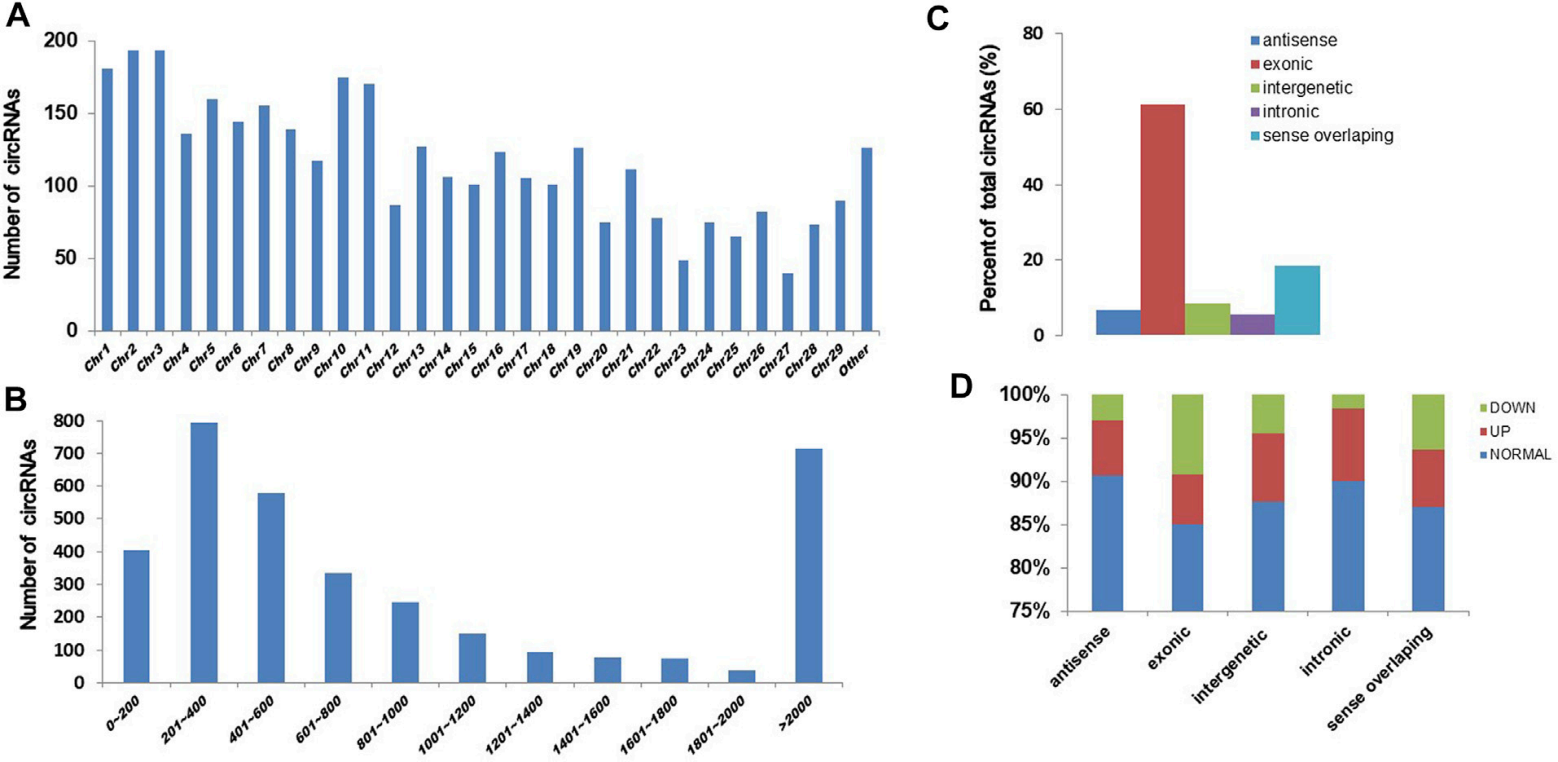
Expression pattern of circRNAs in the GO-AgNPs treated caprine fetal fibroblast cells (CFFCs). **(A)** Distribution of circRNAs on chromosomes. The x-axis represents the chromosome and the y-axis represents the number of circRNA. **(B)** The length distribution of total circRNAs. The x-axis represents the number of circRNAs and the y-axis represents the number of circRNA. **(C)** The genomic origin of the total circRNAs. **(D)** Percent of differentially expressed circRNAs.

**FIGURE 3:**
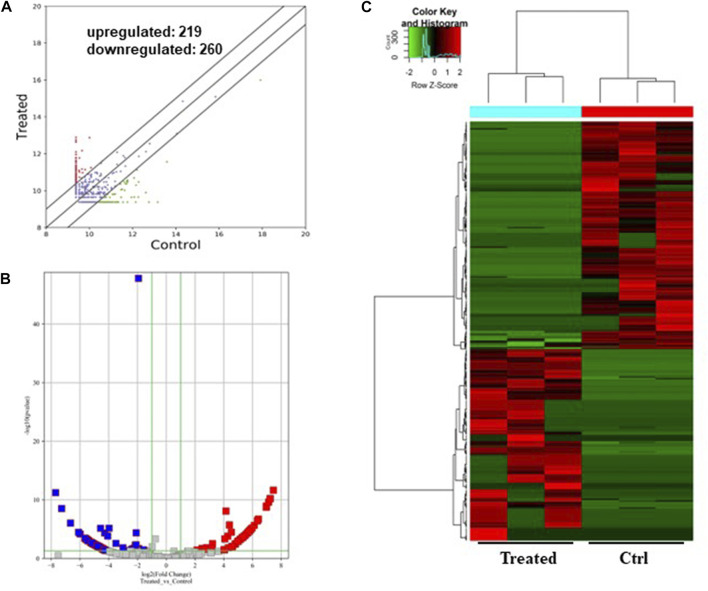
Differentially expressed circRNAs in the GO-AgNPs treated caprine fetal fibroblast cells. **(A)** Hierarchical cluster analysis of circRNAs in the cells between treated and control group. The values of the x and y-axes in the scatterplot are the averaged normalized signal values of groups of samples (log2 scaled). The oblique lines are fold change lines. The circRNAs with the red points (219) and blue points (260) indicate > 2.0 fold changes between control and treated cells. **(B)**. Volcano plots were constructed using fold-change values and *p*-values. The red and blue points represent the differentially up- and downregulated circRNAs with statistical significance (fold-changes of > 2 and *p* values of < 0.05). **(C)** Clustered heatmap of differentially expressed circRNAs in the cells. The red and green represent upregulated and downregulated circRNAs, respectively.

To know the effect of chronic exposure to GO-AgNPs (0.5 μg/mL for 10 weeks) on circRNA expression, the RNA samples obtained from both treated and non-treated CFFCs were sequenced using Illumina high throughput sequencing platform. A total of 3,508 circRNAs were identified in CFFCs, which have not been reported before. As shown in Figure 2, most of the circRNAs were located on chromosomes 1–29 (Figure 2A) and the size of the detected circRNAs ranged between 62 and 90,000 nucleotides (Figure 2B). Although most of the circRNAs were of exonic origin (Figure 3C) but there were also circRNAs that were originated from non-exonic genome such as antisense, intergenetic and intronic (Figure 2D).

### Differentially expressed circRNAs in GO-AgNPs treated CFFCs

As shown in Figure 3, a total of 479 circRNAs were expressed differentially between the control and treated groups: 219 upregulated and 260 downregulated (Figures 3A, B). Hierarchical clustering showed the circRNA expression pattern in the 0.5 μg/mL GO-AgNPs treated and non-treated CFFCs (Figure 3C).

### Different biological processes and signaling pathways in GO-AgNPs treated CFFCs

**FIGURE 4:**
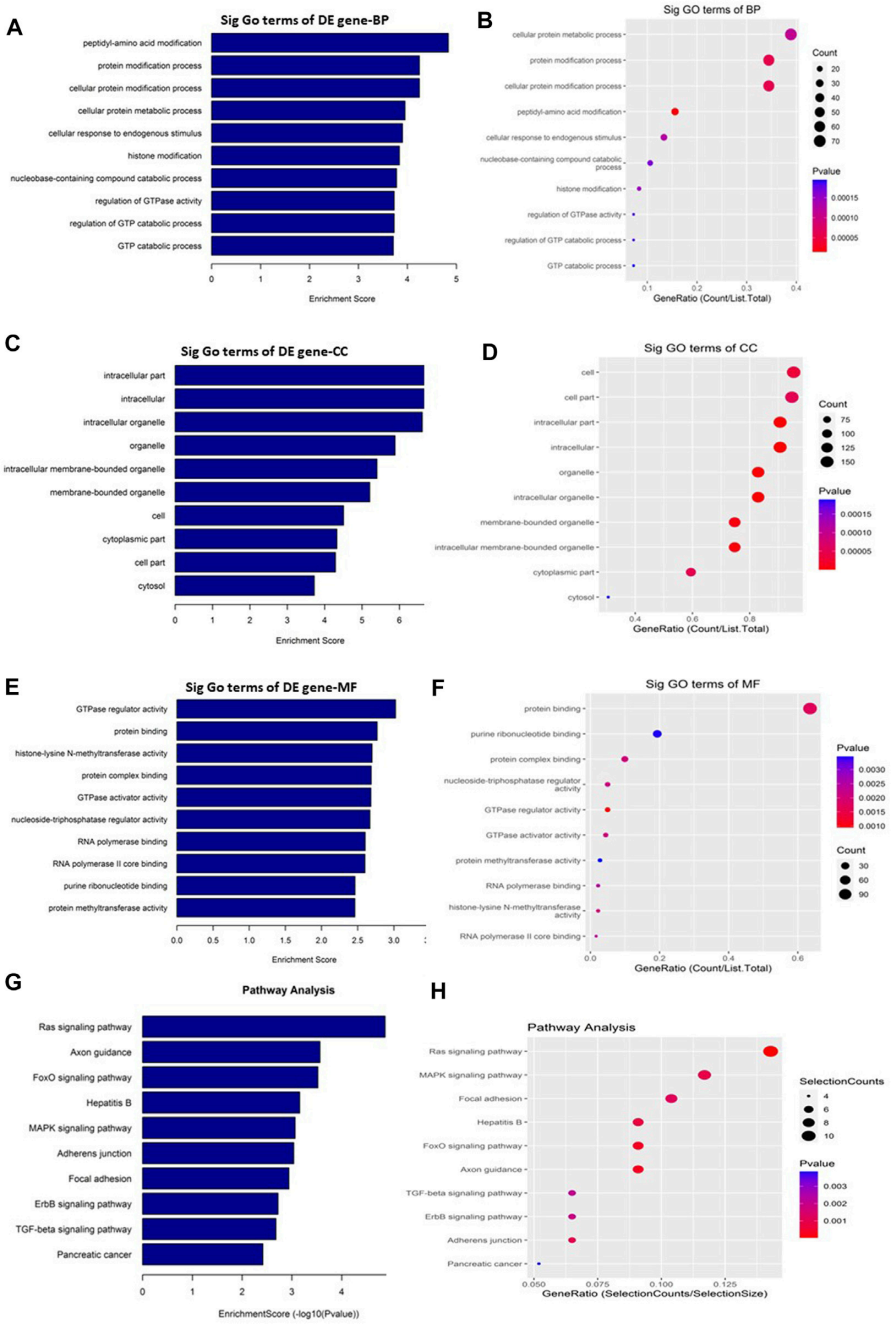
Gene Ontology (GO) and Kyoto Encyclopedia of Genes and Genomes (KEGG) pathway analyses of downregulated circRNAs in the GO-AgNPs treated caprine fetal fibroblast cells (CFFCs). GO analysis identified biological process (BP), cellular component (CC) and molecular function (MF) with the enrichment score [(**A,C,E)** respectively] and gene count **(B,D,F)**. The top ten enriched KEGG pathways of significantly altered circRNA genes **(G,H)**. The color intensity of the nodes shows the degree of enrichment of this analysis. The enrich-factor is defined as the ratio of the differential genes in the entire genome. The dot size represents the count of genes in a pathway.

GO and KEGG pathway enrichment analyses were performed to understand the biological roles of the DE circRNAs. GO analysis showed that the downregulated circRNAs are mainly involved in peptidyl-amino acid modification, intracellular, and GTPase regulator activity (Figures 4A–F) while the KEGG pathway enrichment indicated that the downregulated circRNAs are predominantly connected to the Ras signaling pathway (Figures 4G, H). The other KEGG pathways that are potentially connected to the downregulated circRNAs includes Axon guidance, FoxO signaling pathway, Hepatitis B, MAPK signaling pathway, Adherens junction, Focal adhesion, ErbB signaling pathway, TGF-beta signaling pathway, and Pancreatic cancer.

**FIGURE 5:**
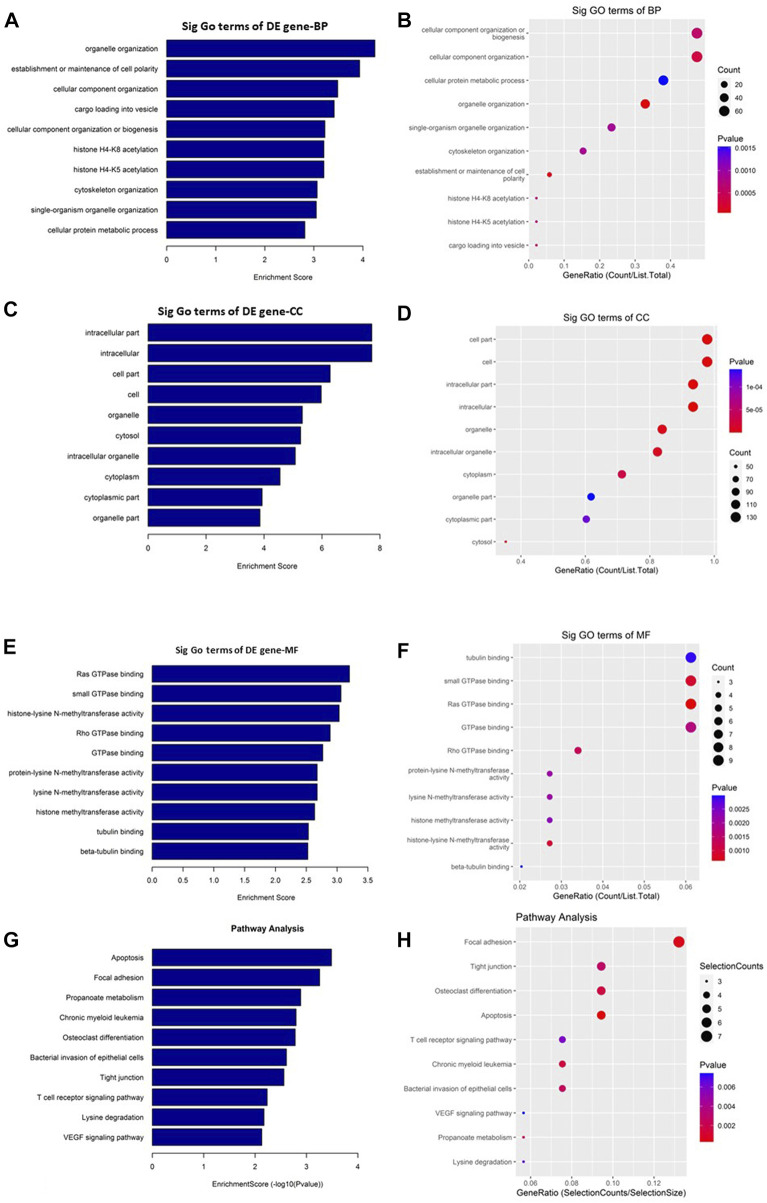
Gene Ontology (GO) and Kyoto Encyclopedia of Genes and Genomes (KEGG) pathway analyses of upregulated circRNAs in the GO-AgNPs treated caprine fetal fibroblast cells (CFFCs). GO analysis identified biological process (BP), cellular compo-nent (CC) and molecular function (MF) with the enrichment score [**(A,C,E)** respectively] and gene count **(B,D,F)**. The top ten enriched KEGG pathways of significantly altered circRNA genes **(G,H)**. The color intensity of the nodes shows the degree of enrichment of this analysis. The enrich-factor is defined as the ratio of the differential genes in the entire genome. The dot size represents the count of genes in a pathway.

On the other hand, the upregulated circRNAs showed involvement mainly in organelle organization, intracellular part and ras GTPase binding (Figures 5A–F). They are also potentially involved in the epigenetic regulation through histone H4K8 and H4K5 acetyla-tion. In addition, the upregulated circRNAs showed high enrichment for KEGG apoptosis pathway (Figures 5G, H). Among the other KEGG pathways that showed enrichment for circRNAs includes focal adhesion, propanoate metabolism, chronic myeloid leukemia, osteoclast differentiation, bacterial invasion of epithelial cells, tight junction, T cell recep-tor signaling pathway, lysine degradation, and VEGF signaling pathway.

### Differential expression of transcripts in GO-AgNPs treated CFFCs

**FIGURE 6:**
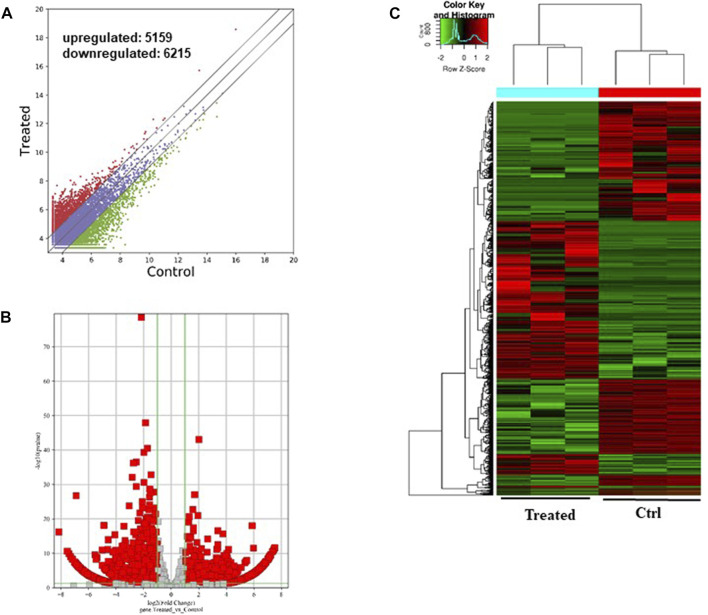
Differentially expressed mRNAs in the GO-AgNPs treated caprine fetal fibroblast cells (CFFCs). **(A)** Hierarchical cluster analysis of mRNAs in the cells. The mRNAs with the red points and blue points indicate > 2.0 fold changes between control and treated cells. **(B)** Volcano plots were constructed using fold-change values and *p*-values. The red and blue points represent the differentially up- and downregulated mRNAs with statistical significance (fold-changes of > 2 and *p*-values of < 0.05). **(C)** Clustered heatmap of differentially expressed mRNAs in the cells.

As shown in Figure 6, a total of 11374 messenger RNAs (mRNAs) were differentially expressed in the CFFCs where 5,159 mRNAs upregulated, and 6,215 mRNAs downregulated (Figures 6A, B). The hierarchical cluster showed the differential expression pattern of mRNAs between 0.5 μg/mL GO-AgNPs treated and non-treated CFFCs (Figure 6C).

### Different biological processes and signaling pathways

**FIGURE 7:**
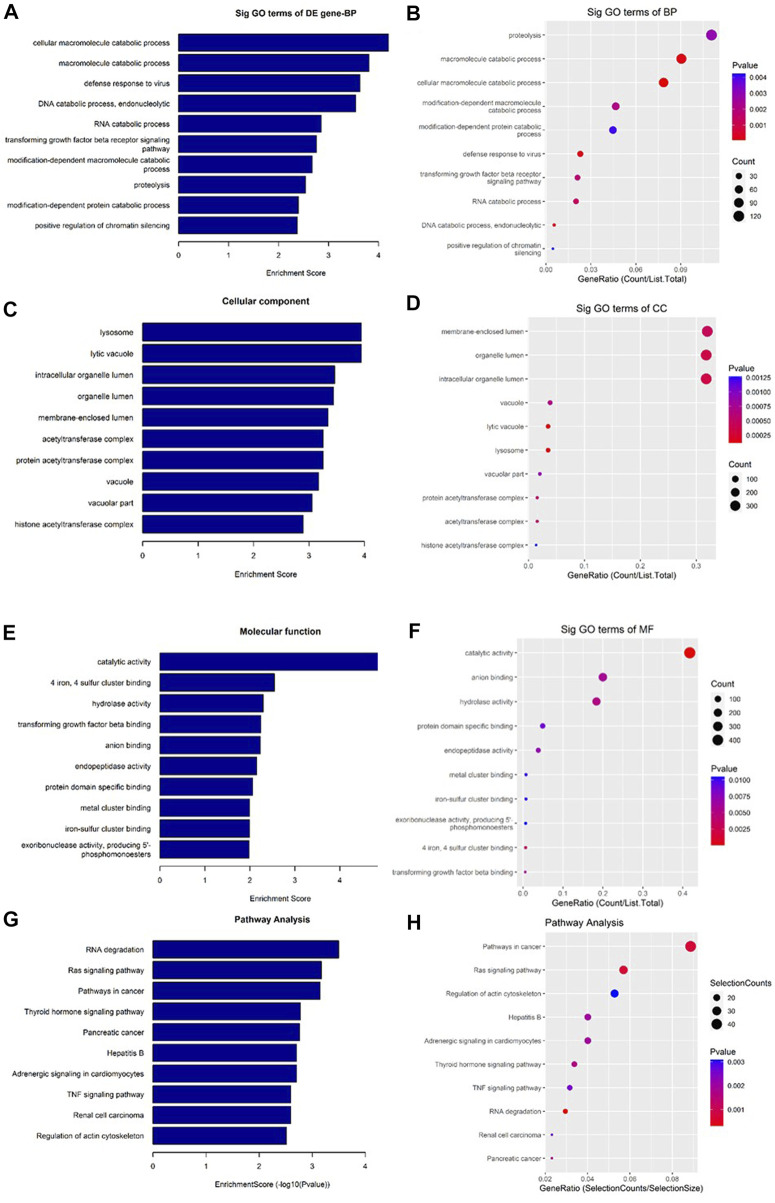
Gene Ontology (GO) and Kyoto Encyclopedia of Genes and Genomes (KEGG) pathway analyses of downregulated mRNAs in the GO-AgNPs treated caprine fetal fibroblast cells (CFFCs). GO analysis identified biological process (BP), cellular component (CC) and molecular function (MF) with the enrichment score [**(A,C,E)** respectively] and gene count **(B,D,F)**, The top ten enriched KEGG pathways of significantly altered mRNA genes **(G,H)**. The color intensity of the nodes shows the degree of enrichment of this analysis. The enrich-factor is defined as the ratio of the differential genes in the entire genome. The dot size represents the count of genes in a pathway.

**FIGURE 8:**
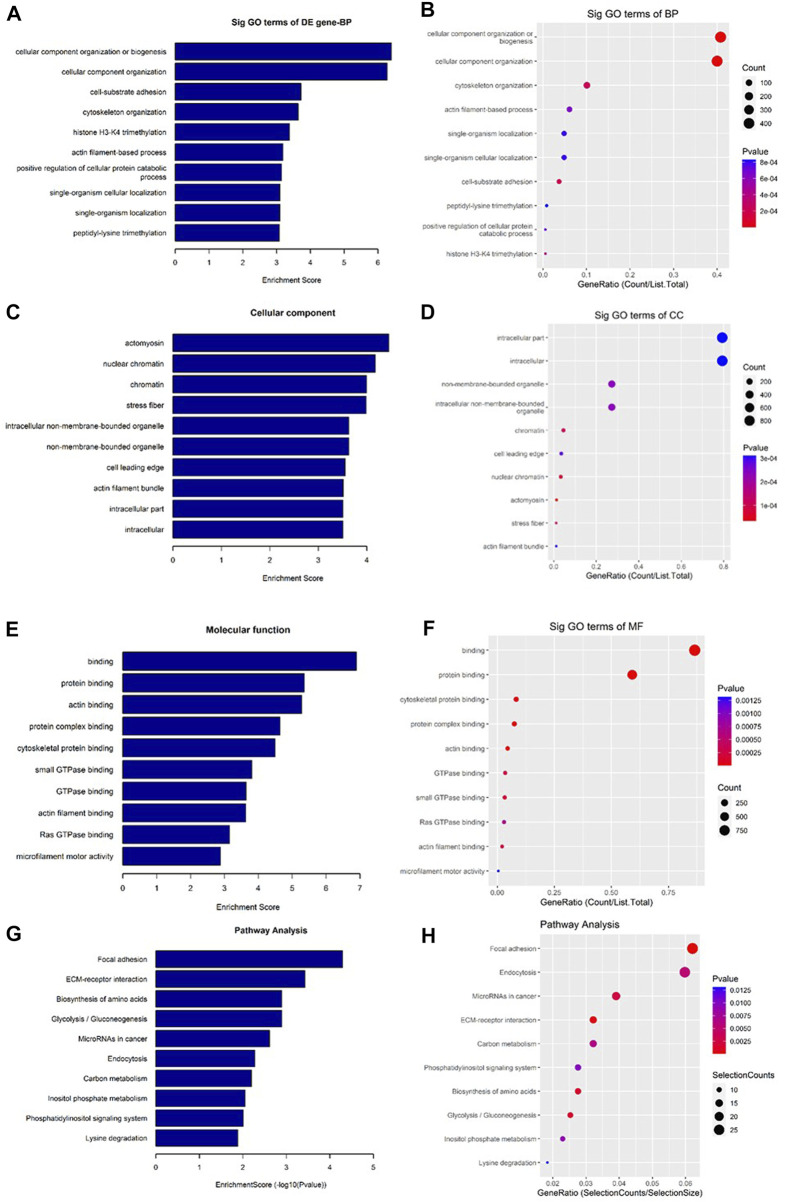
Gene Ontology (GO) and Kyoto Encyclopedia of Genes and Genomes (KEGG) pathway analyses of upregulated mRNAs in the GO-AgNPs treated caprine fetal fibroblast cells (CFFCs). GO analysis identified biological process (BP), cellular component (CC) and molecular function (MF) with the enrichment score [**(A,C,E)** respectively] and gene count **(B,D,F)**. The top ten enriched KEGG pathways of significantly altered mRNA genes **(G,H)**. The color intensity of the nodes shows the degree of enrichment of this analysis. The enrich-factor is defined as the ratio of the differential genes in the entire genome. The dot size represents the count of genes in a pathway.

As shown in Figure 7, the downregulated transcripts have potential regulatory roles in cellular macromolecule catabolic process, lysosome formation, and catalytic activity (Figures 7A–F). In addition, these downregulated transcripts might have important role in the signaling pathways related to cancer (Figures 7G, H). On the other hand, the upregu-lated mRNAs are potentially involved in the biogenesis and organization of cellular components such as actomyosin (Figures 8A–F) as well as signaling pathways related to focal adhesion, ECM-receptor interaction, and biosynthesis of amino acids (Figures 8G, H).

### Validation of RNA-sequence data by qRT-PCR

**FIGURE 9:**
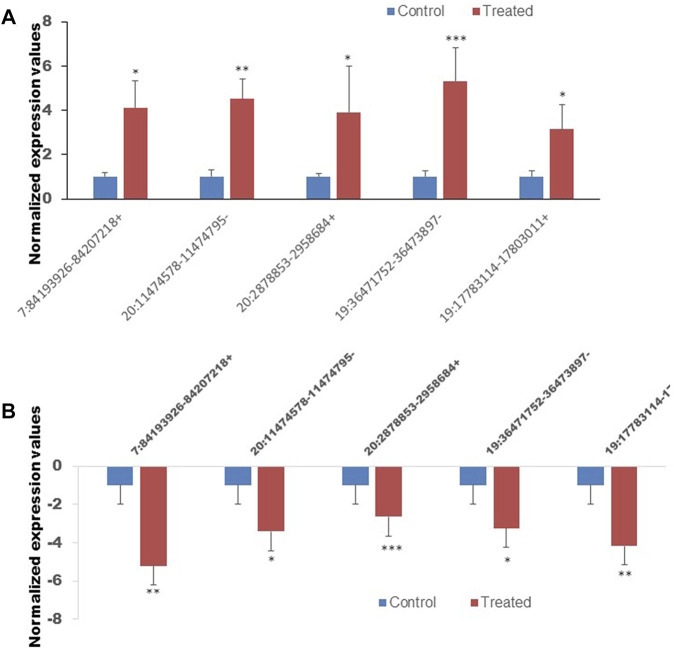
The expression of ten circRNAs confirmed by the quan-titative real-time reverse transcription-polymerase chain reaction (qRT-PCR). **(A)** The expression of five selected upregulated circRNAs. **(B)** The expression of five selected downregulated circRNAs. *p < 0.05, **p < 0.01, ***p < 0.001. The primer sets that were used to amplify the selected circRNAs are enlisted in Table 1.

To confirm the reliability of the sequencing results of top differentially expressed circRNAs (Tables 2, 3), the expression level of 10 DE circRNAs (five upregulated and five downregulated) were tested by qRT-PCR. The results showed similar pattern of circRNAs expression as it was initially detected by high throughput sequencing (Figure 9).

**TABLE 2:** Top 10 significantly downregulated circRNAs.

CircRNAID	logFC	logCPM	F	*p*-value	FDR	Chrom	Source	Catalog
17:9759064-9766303+	−7.6884	12.33191	47.37877	6.lE-12	7.12E-09	17	Novel	Intronic
2:130861551-130862388+	−7.271	11.98669	35.13629	3.15E-09	l.22E-06	2	Novel	Exonic
8:90045854-90066768+	−6.65607	11.5076	24.06098	9.44E-07	0.000236	8	Novel	Exonic
4:33600215-33618663+	−6.09751	11.10789	17.09744	3.57E-05	0.004812	4	Novel	Exonic
11:29744772-29745680−	−5.98737	11.03373	15.85853	6.86E-05	0.007752	11	Novel	Exonic
5:62814807-62823648−	−5.65632	10.82104	12.7958	0.000349	0.025438	5	Novel	Exonic
2:122905686-122911760+	−5.63859	10.80995	12.66566	0.000374	0.026713	2	Novel	Exonic
5:43950436-43963546−	−5.61539	10.7955	12.49752	0.000409	0.028641	5	Novel	Exonic
12:74110524-74125943−	−5.59583	10.78381	12.31825	0.00045	0.030906	12	Novel	Exonic
2:73216739-73217690+	−5.57488	10.77088	12.16547	0.000488	0.031677	2	Novel	Sense overlapping

**TABLE 3:** Top 10 upregulated circRNAs.

CircRNAID	logFC	logCPM	F	*p*-value	FDR	Chrom	Source	Catalog
7:84193926-84207218+	7.453771	11.98739	49.26479	2.34E-12	4.lE-09	7	Novel	Exonic
20:11474578-11474795−	7.239814	11.8019	42.90522	5.95E-11	4.89E-08	20	Novel	Intronic
20:2878853-2958684+	7.226138	11.79123	42.591 13	6.98E-l l	4.89E-08	20	Novel	Sense overlapping
19:36471752-36473897−	7.127393	11.70576	40.11174	2.47E-10	l.44E-07	19	Novel	Exonic
19:17783114-17803011+	6.977031	11.58162	37.09978	l .15E-09	5.76E-07	19	Novel	Exonic
1:138327029-138335074−	6.966996	11.57406	36.54175	l.91E-09	8.36E-07	1	Novel	Exonic
25:26123127-26123621+	6.414496	11.13445	27.06774	l .99E-07	6.34E-05	25	Novel	Antisense
7:95415786-95417595+	6.350182	11.08637	25.89975	3.64E-07	0.000106	7	Novel	Exonic
5:56208942-56209543−	6.339518	11.07899	25.73752	3.96E-07	0.000107	5	Novel	Intergenic
26:10158184-10175290+	6.130048	10.92732	22.37917	2.26E-06	0.000495	26	Novel	Exonic

### CircRNA-miRNA-mRNA network analysis and related prediction

**FIGURE 10:**
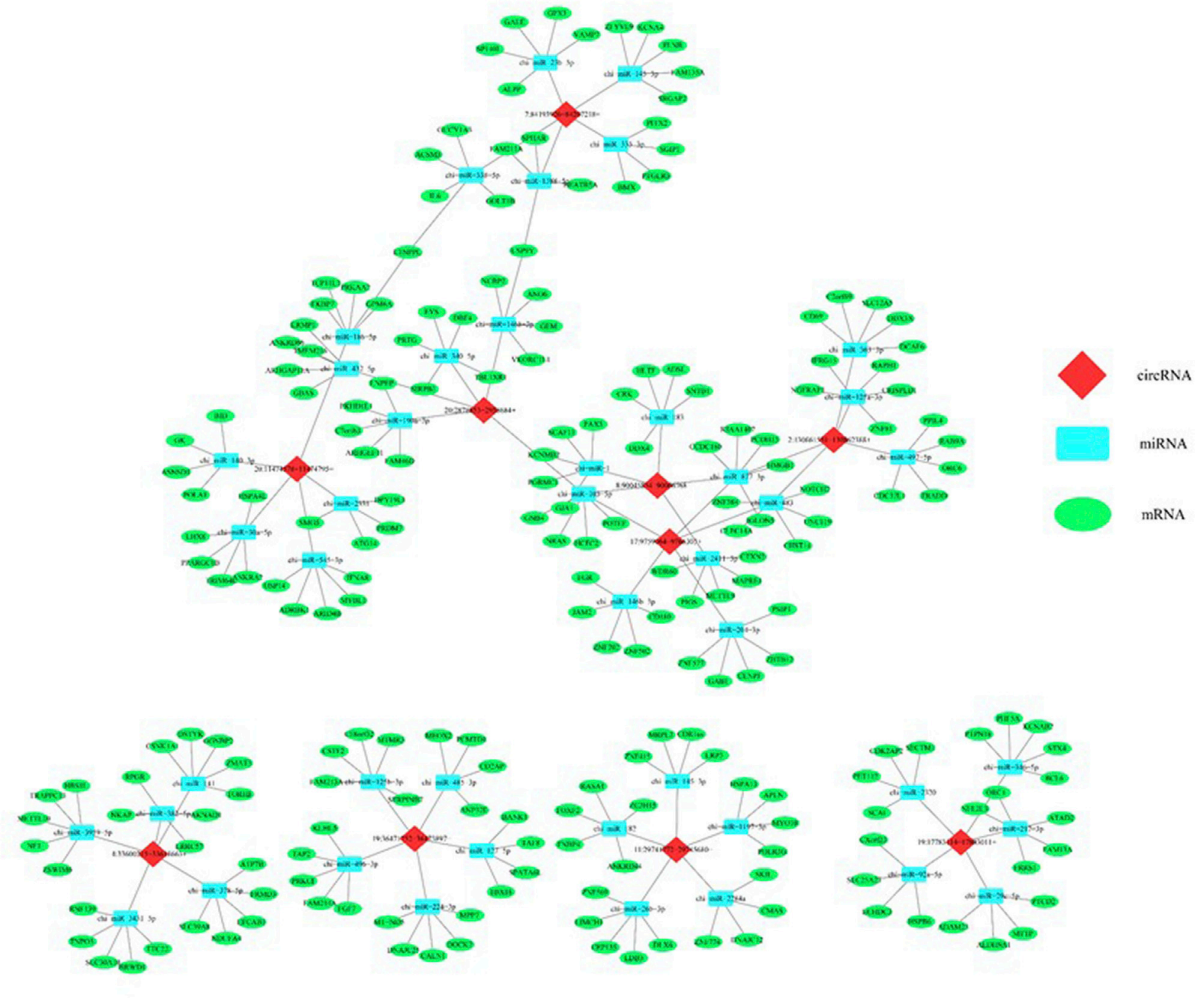
In silico analysis predicted a regulatory network consisted of circRNAs, miRNAs, and mRNAs that potentially regulates the epigenetic toxicity in caprine fetal fibroblast cells (CFFCs) induced by the chronic exposure to the GO-AgNPs. Each of the top five circRNAs related to five miRNAs and mRNAs were selected to construct the network.

Five confirmed circRNAs were considered to predict their regulatory network using bioinformatics analysis. As shown in Figure 10, all five circRNAs are connected to the miRNAs that play regulatory roles on the differentially expressed mRNAs. This indicates that the circRNA-miRNA-mRNA creates an intimate regulatory loop.

## Discussion

CircRNAs is a large class of non-coding RNA which has regulatory roles on different molecules including mRNAs, non-coding RNAs, DNAs and proteins (Chen, 2020) as well as on various biological processes (Huang et al., 2020; Yang et al., 2021). To understand how nanotoxicity interferes the ex-pression pattern and functions of circRNAs, we have induced GO-AgNPs mediated chronic toxicity in CFFCs. This does not cause oxidative stress and cell death but resulted in differential expression of mRNAs and circRNAs. Further, the *in silico* analysis predicted that these differentially expressed circRNAs and mRNAs along with the miRNAs creates a regulatory loop to induce the GO-AgNPs mediated epigenetic toxicity in CFFCs.

The unaltered cell viability and ROS level upon treatment with 0.5 μg/mL GO- AgNPs for 10 weeks has ensured that the experimental does was indeed non-cytotoxic for CFFCs. Previous experiment showed that human dermal fibroblast remains viable up to 20 weeks under the exposure of gold nanoparticles (Falagan-Lotsch et al., 2016) while HaCaTs have insignificant change in cell viability after 14 weeks’ exposure of silver nanoparticles (Comfort et al., 2014). Long-term exposure to AgNPs also induces cell transformation (Choo et al., 2016; Vila et al., 2017). Therefore, our results along with the previous findings suggest that a chronic exposure to different nanoparticles could be visibly non-cytotoxic and non-apoptotic. Of course, an acute dose of nanoparticle is always cytotoxic, as we have shown that only 24 h exposure to 4–8 μg/mL GO-AgNPs is detrimental to the CFFCs (Yuan et al., 2021). Although, the cytotoxicity varies among the type of cells (Yuan and Gurunathan, 2017b; Yuan et al., 2017c), the graphene originated nanomaterials mediated toxicity is well proven in both animal and human cells (Ema et al., 2017a; Wu et al., 2021), and showed much more activity against cancerous cells than normal ones by increasing the expression level of the intracellular ROS and Bax/Bcl-2 genes (Gurunathan et al., 2019).

Accumulated evidence demonstrates that epigenetic modification have been used to check the toxicity inducing by engineered nanomaterials and nanoparticles and, more importantly, to predict their toxicity in preclinical assessments. Several types of nanoparticles have been proved to induce epigenetic modification in DNA methylation patterns, histone modifications and miRNA expressions (Wong et al., 2017). The expression of miRNAs was significantly altered by treatment with graphene oxide or AgNPs, which involved in various apoptosis-related biological pathways (Brzóska et al., 2019; Gurunathan et al., 2019). However, how the chronic exposure to na-noparticles changes epigenetic signature of the cells is not well understood. In the present research, we have found that even a visibly non-cytotoxic and non-apoptotic dose of GO-AgNPs can significantly alter the epigenetic status of CFFCs. The sequencing data showed that 479 circRNAs were differentially expressed in CFFCs after exposed to 0.5 μg/mL GO-AgNPs, of which 219 genes were upregulated and 260 genes were downregulated. 5,159 mRNAs were upregulated and 6,215 mRNAs were downregulated. Interestingly, the altered circRNAs are connected to a group of miRNAs (such as miR-186-5p, miR432-5p, miR-190a-3p) that potentially regulate the expression of the deregulated mRNAs. Further, our *in silico* prediction showed that several signaling pathways and biological processes are associated with the differentially expressed circRNAs. This means that the circRNAs, miRNAs and mRNAs created a regulatory loop to response the chronic toxicity induced by GO-AgNPs. It is known that different nanoparticles can alter the expression of genes (Ganjouzadeh et al., 2022), and miRNAs (Eom et al., 2014; Oh et al., 2016). But the alteration of circRNAs expression in response to the chronic exposure to the GO-AgNPs has not been reported yet. Hence, our findings revealed the GO-AgNPs induced alteration of circRNAs expression in CFFCs and its potential relevance to the biological processes.

Moreover, previous researches have shown that circRNAs regulate epigenetic status by mediating histone modification in response to both *in vivo* and *in vitro* exposure of nanomaterials (Pogribna and Hammons, 2021). Treatment of mouse erythroleukemia cells or A549 cells with 8 or 10 μg/mL AgNPs modified level of acetylation of histone H3 (Qian et al., 2015; Blanco et al., 2017). Our GO results showed that the differentially expressed circRNAs are potentially connected to a group of genes that are related to epigenetic modifications such as histone modification, histone-lysine N-methyltransferase activity, histone H4K5/8 acetylation, lysine N-methyltransferase activity and histone methyltransferase activity while the genes that are connected to the DE circRNAs includes KAT7, SMYD3, KMT2C/D and TET3, which are related to histone modification. CircMRPS35 can recruit KAT7 to the promoters in FOXO1 and FOXO3a genes, which elicits acetylation of H4K5 in their promoters (Jie et al., 2020). SMYD3 and KMT2C/D is histone H3 lysine 4 dimethyltransferase and trimethyltransferase (Hamamoto et al., 2004; Allis et al., 2007), resulting in a significant reduction of global methylation for gene activation (Qian et al., 2015). Previous studies demonstrated that the reduced level of global DNA methylation by nanoparticle were accompanied by a decrease in total activity of DNA methyltransferase such as DNMT, TET (Patil et al., 2016; Choudhury et al., 2017). Overall, our data may highlight the cross-talk between histone modification and toxicity induced by nanoparticle.

The interaction between the top five up- and five downregulated circRNAs and their respected 5 miRNA targets were predicted by conserved seed-matching sequencing using TargetScan and miRanda database, and Cytoscape software was used to construct a network. The circRNA-miRNA network indicated that all of the DE circRNAs have their respective miR response elements, which may provide direction for further studies.

## Conclusion

In the present study, 0.5 μg/mL GO-AgNPs did not increase intracellular ROS production in CFFCs after tested for 10 weeks but induced epigenetic alterations. Enriched GO terms and KEGG pathways for differentially circRNAs showed involvement mainly in organelle organization, intracellular part, ras GTPase binding and the Ras signaling pathway. In conclusion, chronic exposure of GO-AgNPs causes epigenetic alterations in CFFCs that may be potentially controlled by a network consisted of circRNAs, miRNAs, and mRNAs. However, further studies are required to understand whether alteration of circRNAs expression is the ultimate cause of epigenetic changes or it is just another component of the regulatory loop. Future studies may focus on understanding of the mechanisms and processes in epigenetic alterations induced by the nano-materials and the role of epigenetic alterations as biomarkers of the nanomaterials’ toxicity. The role of special circRNA in governing histone modification also need to be further identified in the GO-AgNPs treated CFFCs.

## Data Availability

The datasets presented in this study can be found in online repositories. The names of the repository/repositories and accession number(s) can be found below: https://www.ncbi.nlm.nih.gov/, GSE199594.
